# Improved Iterative Calibration for Triaxial Accelerometers Based on the Optimal Observation

**DOI:** 10.3390/s120608157

**Published:** 2012-06-12

**Authors:** Jie Yang, Wenqi Wu, Yuanxin Wu, Junxiang Lian

**Affiliations:** College of Mechanical Engineering and Automation, National University of Defense Technology, Changsha 410073, China; E-Mails: nudtyang@163.com (J.Y.); wenqiwu_lit@hotmail.com (W.W.); jx_lian@yahoo.com.cn (J.L.)

**Keywords:** triaxial accelerometers, maximum likelihood estimation, iterative calibration, nonlinear, optimization

## Abstract

This paper presents an improved iterative nonlinear calibration method in the gravitational field for both low-grade and high-grade triaxial accelerometers. This calibration method assumes the probability density function of a Gaussian distribution for the raw outputs of triaxial accelerometers. A nonlinear criterion function is derived as the maximum likelihood estimation for the calibration parameters and inclination vectors, which is solved by the iterative estimation. First, the calibration parameters, including the scale factors, misalignments, biases and squared coefficients are estimated by the linear least squares method according to the multi-position raw outputs of triaxial accelerometers and the initial inclination vectors. Second, the sequence quadric program method is utilized to solve the nonlinear constrained optimization to update the inclination vectors according to the estimated calibration parameters and raw outputs of the triaxial accelerometers. The initial inclination vectors are supplied by normalizing raw outputs of triaxial accelerometers at different positions without any *a priori* knowledge. To overcome the imperfections of models, the optimal observation scheme is designed according to some maximum sensitivity principle. Simulation and experiments show good estimation accuracy for calibration parameters and inclination vectors.

## 1. Introduction

Triaxial accelerometers have been used extensively in the fields of inertial navigation and gravimetry [1,2]. For accurate specific force measurements, calibration must be implemented to estimate some parameters which transform the raw outputs of accelerometers into linear acceleration. The calibration parameters contain the scale factors, misalignments, biases, nonlinear coefficients, temperature drifts and so on. Traditionally, the calibration relies on some precise inertial test setup to estimate the parameters according to the input and output reference information [2]. However, such an expensive setup is not suitable for low-cost MEMS accelerometers. Meanwhile, nowadays the calibration setup cannot provide enough accurate reference information for high-grade accelerometers due to the unmatched accuracy improvement. Thus an efficient calibration for both low-grade and high-grade triaxial accelerometers needs to be designed without the requirement of precise orientation information.

In recent years, a promising multi-position calibration for triaxial accelerometers in the gravitational field has been proposed as an effective solution to relax the precise orientation supplied by the setup [3–14]. These calibration methods have been implemented based on the fact that the norm of the raw outputs of triaxial accelerometers ideally is equal to the gravity value. In most cases, a cost function, namely, the squared error between the magnitude of input specific force and the magnitude of raw outputs has been utilized to estimate the calibration parameters [3–12]. Different estimation methods have been utilized to attack the optimization problem. In [3] a Newton's iterative method was used to minimize the cost function to get the calibration parameters. In [4] the minimization of the cost function was numerically performed using the lsqnonlin function of the Matlab optimization toolbox. In [5] the downhill simplex optimization method was used to minimize the cost function. In [6–11] the authors utilized the iterative least square estimation method to implement the cost function's minimization. In particular, the authors in [10] argued the calibration improvement and alignment properties of the proposed algorithm. Interestingly, three different calibration strategies for two three-axis sensors are investigated for in-the-field calibration purposes in [11]. In [12] a Kalman filter was used to estimate the calibration parameters. Thus we see that iterative methods are mostly utilized to solve the optimal functions and achieve high estimation accuracy, but the need for an initial rough estimate makes them inconvenient. In [13] a simple non-iteration method without any initial guess has been proposed but the misalignments of triaxial accelerometers are not considered. In [14] the authors summarized the minimization of the cost function as a 3-D ellipsoid-fitting problem and proposed a minimum-volume enclosing ellipsoid optimization to solve the calibration procedure issue. The authors in [15] proposed an improved multi-position calibration for solving the unknown parameters including scale factors, misalignments and biases. However, nonlinear errors have not been considered in the above calibration methods. The authors in [16] solve the calibration problems using the maximum likelihood estimation (MLE) method and validate the asymptotically unbiased property by comparing the variance of estimated parameters with the Cramer-Rao bound. However, some problems are still not settled for the proposed MLE method. Firstly, the initial calibration parameters for the two-step iterative estimation have not been solved. Secondly, the Euler angles representation of the inclination vector is not perfect for the singularity of roll angles as pitch angles approach to 90 degrees or −90 degrees. Thirdly, the optimal observation scheme for the estimation is not discussed. Finally, the nonlinear errors of triaxial accelerometers are not considered.

In view of the above disadvantages, this paper proposes an improved iterative calibration method for both linear and nonlinear models of triaxial accelerometers. For the inclination vector estimation, the second-column elements of direction cosine matrix have been chosen instead of Euler angles to overcome the singularity. Besides, the rough inclination vector estimation at different positions is derived by normalizing the raw outputs of triaxial accelerometers without any *a priori* knowledge. Thus a modified two-step iterative estimation has been designed compared with the estimation flow in [16]. Meanwhile the sequence quadric program (SQP) method is utilized to attack the nonlinear constrained optimal problem in the iterative estimation. For the optimal observation, a maximum sensitivity of some constant output as a function of calibration parameters is designed to make the measurement accuracy of triaxial accelerometers consistent in the whole gravitational field.

This paper is organized as follows: Section 2 describes the linear and nonlinear models of triaxial accelerometers. Section 3 presents the improved iterative calibration method, including the two-step iterative estimation flow and the method to derive initial inclination vectors. The optimal observation in the gravitational field has been designed according to the maximum sensitivity principle in Section 4. Section 5 reports the error analysis by Monte Carlo simulations. Section 6 describes the calibration results for triaxial quartz accelerometers. Meanwhile, the experiment results validate the measurement accuracy improvement by the nonlinear model over the linear model. Conclusions are drawn in Section 7.

## 2. Modeling of Triaxial Accelerometers

### 2.1. Definition of Related Parameters

Some related definitions including the frames and parameters are listed in Table 1.

### 2.2. Linear and Nonlinear Models for the Triaxial Accelerometers

The imperfect installation makes the sensitivity axes of triaxial accelerometers non-orthogonal. For specific force measurements, an orthogonal frame needs to be defined according to some reference information. Here, we define the *b*-frame so that *x_b_* coincides with the sensitivity axis *x_a_, y_b_* lines in the *x_a_y_b_* plane and *z_b_* constitutes a right-handed orthogonal frame with *x_b_* and *y*_b_ Thus, the linear model of triaxial accelerometers can be derived as follows [3–16]:

(1) $$\text{p}={\text{k}}_{\text{a}}T_{b}^{a}\;{\text{f}}^{b}+{\text{p}}_{0}+{\text{v}}_{a}$$

and the corresponding parameters in Equation (1) take the following forms:

(2) $$\text{p}=\left[\begin{array}{l} {\text{p}}_{x}\\ p_{y}\\ p_{z} \end{array}\right],{\text{k}}_{\text{a}}=\left[\begin{array}{ccc} k_{a,x} & 0 & 0\\ 0 & k_{a,y} & 0\\ 0 & 0 & k_{a,z} \end{array}\right],T_{b}^{a}=\left[\begin{array}{ccc} 1 & 0 & 0\\ \tau_{\mathrm{yx}} & \tau_{\mathrm{yy}} & 0\\ \tau_{\mathrm{zx}} & \tau_{\mathrm{zy}} & \tau_{\mathrm{zz}} \end{array}\right],{\text{f}}^{b}=\left[\begin{array}{l} f_{x}^{b}\\ f_{y}^{b}\\ f_{z}^{b} \end{array}\right],p_{0}=\left[\begin{array}{l} p_{0,x}\\ p_{0,y}\\ p_{0,z} \end{array}\right],{\text{v}}_{a}=\left[\begin{array}{l} v_{a,x}\\ v_{a,y}\\ v_{a,z} \end{array}\right]$$

For simple analysis, the measurement noise ***v****_a_* is assumed to be the zero-mean Gaussian white noise with the variance of σ^2^. But the linear model cannot always fit in the precise specific force measurement because of such errors as nonlinearities and temperature drifts. So a nonlinear model including the squared coefficients is derived below [17,18]:

(3) $$\text{p}={\text{k}}_{\text{a}}T_{b}^{a}{\text{f}}^{b}+{\text{p}}_{0}+k_{2}{\text{f}}^{2,b}+{\text{v}}_{a}$$

where:

(4) $${\text{f}}^{2,b}={\left[\begin{array}{ccc} {\left(f_{x}^{b}\right)}^{2} & {\left(f_{y}^{b}\right)}^{2} & {\left(f_{z}^{b}\right)}^{2} \end{array}\right]}^{T},k_{2}=\text{diag}\left(\left[\begin{array}{ccc} k_{2,x} & k_{2,y} & k_{2,z} \end{array}\right]\right)$$

The calibration for both linear and nonlinear models is implemented to estimate the scale factors, misalignments, biases and squared coefficients. At the same time, the specific force of ***f****^b^* can also be estimated. Especially, the observation information including only the raw outputs of triaxial accelerometers and gravity value makes the calibration procedure difficult. An improved iterative calibration method will be described in the following section.

## 3. The Improved Iterative Calibration for the Triaxial Accelerometers

Firstly, an improved two-step iterative calibration algorithm is designed. Secondly, the initial value is supplied according to the raw outputs of triaxial accelerometers.

### 3.1. Improved Two-Step Iterative Estimation Scheme

In the gravitational field, the specific force vector is equal to the minus gravity vector. We define the local level frame, *n*-frame, so that *x_n_* points to north, *y_n_* points to upward, and *z_n_* points to east. Then the gravity vector in *n*-frame can be denoted as **g**^n^ = [0 −g 0]^T^. The relative attitude between *b*-frame and *n*-frame can be represented by the direction cosine matrix of $C_{n}^{b}$. The Euler rotation sequence from *n*-frame to *b*-frame is defined as follows: first around *y*-axis with ψ, then around *z*-axis with θ, and finally around with *x*-axis with *ϕ*, or equivalently:

(5) $$C_{n}^{b}=\left[\begin{array}{ccc} \cos\psi \cos\theta & \sin\theta & -\sin\psi \cos\theta \\ -\cos\psi \sin\theta \cos\phi +\sin\psi \sin\phi & \cos\theta \cos\phi & \sin\psi \sin\theta \cos\phi +\cos\psi \sin\phi \\ \cos\psi \sin\theta \sin\phi +\sin\psi \cos\phi & -\cos\theta \sin\phi & -\sin\psi \sin\theta \sin\phi +\cos\psi \cos\phi \end{array}\right]$$

In the static case, the specific force vector satisfies the following relationship:

(6) $$\begin{array}{cc} {\text{f}}^{b} & =C_{n}^{b}{\text{f}}^{n}=-C_{n}^{b}{\text{g}}^{n}\\ & =\left[\begin{array}{ccc} \cos\psi \cos\theta & \sin\theta & -\sin\psi \cos\theta \\ -\cos\psi \sin\theta \cos\phi +\sin\psi \sin\phi & \cos\theta \cos\phi & \sin\psi \sin\theta \cos\phi +\cos\psi \sin\phi \\ \cos\psi \sin\theta \sin\phi +\sin\psi \cos\phi & -\cos\theta \sin\phi & -\sin\psi \sin\theta \sin\phi +\cos\psi \cos\phi \end{array}\right]\left[\begin{array}{l} 0\\ g\\ 0 \end{array}\right]\\ & =\left[\begin{array}{c} g\sin\theta \\ g\cos\theta \cos\phi \\ -g\cos\theta \sin\phi \end{array}\right]=g\left[\begin{array}{c} c_{1}\\ c_{2}\\ c_{3} \end{array}\right] \end{array}$$

Suppose we have obtained the raw outputs of triaxial accelerometers at *m* positions. Substituting Equation (3) into Equation (6), the observation equation at the *k*-th position is derived as:

(7) $$\begin{array}{ll} \left[\begin{array}{c} p_{x}^{k}\\ p_{y}^{k}\\ p_{z}^{k} \end{array}\right] & =g\left[\begin{array}{ccc} k_{a,x} & 0 & 0\\ 0 & k_{a,y} & 0\\ 0 & 0 & k_{a,z} \end{array}\right]\left[\begin{array}{ccc} 1 & 0 & 0\\ \tau_{\mathrm{yx}} & \tau_{\mathrm{yy}} & 0\\ \tau_{\mathrm{zx}} & \tau_{\mathrm{zy}} & \tau_{\mathrm{zz}} \end{array}\right]\left[\begin{array}{c} c_{1,k}\\ c_{2,k}\\ c_{3,k} \end{array}\right]+\left[\begin{array}{l} p_{0,x}\\ p_{0,y}\\ p_{0,z} \end{array}\right]+g^{2}\left[\begin{array}{c} k_{2,x}c_{1,k}^{2}\\ k_{2,y}c_{2,k}^{2}\\ k_{2,z}c_{3,k}^{2} \end{array}\right]+\left[\begin{array}{l} v_{a,x}\\ v_{a,y}\\ v_{a,z} \end{array}\right]\\ & =g\left[\begin{array}{ccc} k_{a,x} & 0 & 0\\ k_{a,y}\tau_{\mathrm{yx}} & k_{a,y}\tau_{\mathrm{yy}} & 0\\ k_{a,z}\tau_{\mathrm{zx}} & k_{a,z}\tau_{\mathrm{zy}} & k_{a,z}\tau_{\mathrm{zz}} \end{array}\right]\left[\begin{array}{c} c_{1,k}\\ c_{2,k}\\ c_{3,k} \end{array}\right]+\left[\begin{array}{l} p_{0,x}\\ p_{0,y}\\ p_{0,z} \end{array}\right]+g^{2}\left[\begin{array}{c} k_{2,x}c_{1,k}^{2}\\ k_{2,y}c_{2,k}^{2}\\ k_{2,z}c_{3,k}^{2} \end{array}\right]+\left[\begin{array}{l} v_{a,x}\\ v_{a,y}\\ v_{a,z} \end{array}\right]\\ & =g\left[\begin{array}{ccc} k_{\text{axx}} & 0 & 0\\ k_{\text{ayx}} & k_{\text{ayy}} & 0\\ k_{\text{azx}} & k_{\text{azy}} & k_{\text{azz}} \end{array}\right]\left[\begin{array}{c} c_{1,k}\\ c_{2,k}\\ c_{3,k} \end{array}\right]+\left[\begin{array}{l} p_{0,x}\\ p_{0,y}\\ p_{0,z} \end{array}\right]+g^{2}\left[\begin{array}{c} k_{2,x}c_{1,k}^{2}\\ k_{2,y}c_{2,k}^{2}\\ k_{2,z}c_{3,k}^{2} \end{array}\right]+\left[\begin{array}{l} v_{a,x}\\ v_{a,y}\\ v_{a,z} \end{array}\right] \end{array}$$

We can define two sets of vectors from Equation (7), *i.e.*, the parameter vector and inclination vector, as below:

(8) $$\text{x}={\left[\begin{array}{cccccccccccc} k_{\text{axx}} & p_{0,x} & k_{2,x} & k_{\text{ayx}} & k_{\text{ayy}} & p_{0,y} & k_{2,y} & k_{\text{azx}} & k_{\text{azy}} & k_{\text{azz}} & p_{0,z} & k_{2,z} \end{array}\right]}^{T}$$

(9) $$\text{y}={\left[\begin{array}{cccccccccc} c_{1,1} & c_{2,1} & c_{3,1} & c_{1,2} & c_{2,2} & c_{3,2} & \cdots & c_{1,m} & c_{2,m} & c_{3,m} \end{array}\right]}^{T}$$

Thus the nonlinear model of triaxial accelerometers at the *k*-th position can be represented as:

(10) $${\text{p}}_{k}=\mu_{k}\left(\text{x},{\text{y}}_{k}\right)+{\text{v}}_{a}$$

where

(11) $$\mu_{k}\left(\text{x},{\text{y}}_{k}\right)=\left[\begin{array}{l} \mu_{1,k}\\ \mu_{2,k}\\ \mu_{3,k} \end{array}\right]=k_{a}T_{b}^{a}C_{n}^{b}{\text{f}}^{n}+{\text{p}}_{0}+k_{2}{\text{f}}^{2,b}=\left[\begin{array}{c} gk_{\text{axx}}c_{1,k}+p_{0,x}+g^{2}k_{2,x}c_{1,k}^{2}\\ gk_{\text{ayx}}c_{1,k}+gk_{\text{ayy}}c_{2,k}+p_{0,y}+g^{2}k_{2,y}c_{2,k}^{2}\\ gk_{\text{azx}}c_{1,k}+gk_{\text{azy}}c_{2,k}+gk_{\text{azz}}c_{3,k}+p_{0,z}+g^{2}k_{2,z}c_{3,k}^{2} \end{array}\right]$$

Then for *m* sets of positions, we have:

(12) $$\text{p}={\left[{\text{p}}_{1}^{T}\begin{array}{c} {\text{p}}_{2}^{T}\begin{array}{c} \cdots \end{array}\begin{array}{c} {\text{p}}_{m}^{T} \end{array} \end{array}\right]}^{T},\mu \left(\text{x},\text{y}\right)={\left[\begin{array}{cccc} \mu_{1}\left(\text{x},{\text{y}}_{1}\right) & \mu_{2}\left(\text{x},{\text{y}}_{2}\right) & \cdots & \mu_{m}\left(\text{x},{\text{y}}_{m}\right) \end{array}\right]}^{T}$$

For the zero-mean Gaussian white noise, the raw output of ***p*** is subjected to the Gaussian distribution. Thus the following nonlinear least square optimal function can be derived by the maximum likelihood estimation method [16]:

(13) $$\left(\hat{\text{x}},\hat{\text{y}}\right)=\text{arg min}\;J\left(\text{p},\text{x},\text{y}\right)=\text{arg min}\sum_{k=1}^{m}{\left\|{\text{p}}_{k}-\mu_{k}\left(x,y_{k}\right)\right\|}^{2}$$

Considering the nonlinear objection function in Equation (13), a two-step separation estimation method can be utilized below, as described below.

#### 3.1.1. Estimation of the Parameter Vector

Given the inclination vector *ŷ* the parameter vector ***x*** is estimated by the following optimal solution:

(14) $$\hat{\text{x}}=\text{argmin}\;J\left(\text{p},\text{x},\hat{y}\right)=\text{argmin}\sum_{k=1}^{m}{\left\|{\text{p}}_{k}-\mu_{k}\left(x,{\hat{y}}_{k}\right)\right\|}^{2}$$

and the linear observation function can be easily derived from Equation (14) as follows [19,20]:

(15) $$F(\hat{y})\text{x}=\left[\begin{array}{c} \ldots \\ \begin{array}{cccccccccccc} gc_{1,k} & 1 & g^{2}c_{1,k}^{2} & 0 & 0 & 0 & 0 & 0 & 0 & 0 & 0 & 0\\ 0 & 0 & 0 & gc_{1,k} & gc_{2,k} & 1 & g^{2}c_{2,k}^{2} & 0 & 0 & 0 & 0 & 0\\ 0 & 0 & 0 & 0 & 0 & 0 & 0 & gc_{1,k} & gc_{2,k} & gc_{3,k} & 1 & g^{2}c_{3,k}^{2} \end{array}\\ \ldots \end{array}\right]\text{x}=\text{p}=\left[\begin{array}{l} \cdots \\ p_{x}^{k}\\ p_{y}^{k}\\ p_{z}^{k}\\ \cdots \end{array}\right]$$

Thus the parameter vector can be easily estimated according to the multi-position observation.

#### 3.1.2. Estimation of the Combined Inclination Vector

Given the parameter vector *x̂*, the combined inclination vector ***y*** is estimated by solving the following optimal function:

(16) $$\hat{\text{y}}=\text{argmin}\;J\left(\text{p},\hat{x},\text{y}\right)=\text{argmin}\sum_{k=1}^{m}{\left\|{\text{p}}_{k}-\mu_{k}\left(\hat{x},y_{k}\right)\right\|}^{2}$$

As noises at different positions are independent, the solution of combined inclination vector is identical to solving the individual inclination vector. Utilizing the above estimated parameter vector, we can get the following observation equation at the *k*-th position:

(17) $$\begin{array}{c} \begin{array}{l} g\left[\begin{array}{ccc} k_{\text{axx}} & 0 & 0\\ k_{\text{ayx}} & k_{\text{ayy}} & 0\\ k_{\text{azx}} & k_{\text{azy}} & k_{\text{azz}} \end{array}\right]\left[\begin{array}{c} c_{1,k}\\ c_{2,k}\\ c_{3,k} \end{array}\right]+\left[\begin{array}{l} p_{0,x}\\ p_{0,y}\\ p_{0,z} \end{array}\right]+g^{2}\left[\begin{array}{l} k_{2,x}c_{1,k}^{2}\\ k_{2,y}c_{2,k}^{2}\\ k_{2,z}c_{3,k}^{2} \end{array}\right]=\left[\begin{array}{l} p_{x}^{k}\\ p_{y}^{k}\\ p_{z}^{k} \end{array}\right] \end{array}\\ \begin{array}{ll} s.t. & \;c_{1,k}^{2}+c_{2,k}^{2}+c_{3,k}^{2}=1 \end{array} \end{array}$$

Obviously, the above constrained problem is a nonlinear constrained estimation, which can be effectively solved by the sequence quadratic program (SQP) method [21]. Firstly, the standard constrained optimal presentation can be derived from Equation (17) as:

(18) $$\begin{array}{l} \min f\left(c_{k}\right)={\left(gk_{\text{axx}}c_{1,k}+p_{0,x}+g^{2}k_{2,x}c_{1,k}^{2}-p_{x}^{k}\right)}^{2}+{\left(gk_{\text{ayx}}c_{1,k}+gk_{\text{ayy}}c_{2,k}+p_{0,y}+g^{2}k_{2,y}c_{2,k}^{2}-p_{y}^{k}\right)}^{2}+{\left(gk_{\text{azx}}c_{1,k}+gk_{\text{azy}}c_{2,k}+gk_{\text{azz}}c_{3,k}+p_{0,z}+g^{2}k_{2,z}c_{3,k}^{2}-p_{z}^{k}\right)}^{2}\\ s.t.\;h\left(c_{k}\right)=c_{1,k}^{2}+c_{2,k}^{2}+c_{3,k}^{2}-1=0 \end{array}$$

The Lagrange function can be constructed from Equation (18) below:

(19) $$L\left(c_{k},u_{k}\right)=f\left(c_{k}\right)+u_{k}h\left(c_{k}\right)$$

Thus the Karush-Kuhn-Tucker (KKT) condition equation is derived as:

(20) $$\begin{array}{cc} \nabla f\left(c_{k}\right)+u_{k}\nabla h\left(c_{k}\right)=0, & h\left(c_{k}\right)=c_{1,k}^{2}+c_{2,k}^{2}+c_{3,k}^{2}-1=0 \end{array}$$

The first KKT equation in Equation (20) means ∇*L*(*c_k_*,*u_k_*)=0;, which can be solved by the following Newton iterative method:

(21) $$\begin{array}{cc} c_{k,n+1}=c_{k,n}+\delta c_{k}, & u_{k,n+1}=u_{k,n}+\delta u_{k} \end{array}$$

and the correction quantity of σ*c_k_* and σ*u_k_* in Equation (21) is the solution of the following linear equations:

(22) $$-\left(\begin{array}{cc} \nabla_{c_{k}}^{2}L\left(c_{k},u_{k}\right) & \nabla h\left(c_{k}\right)\\ {\left(\nabla h\left(c_{k}\right)\right)}^{T} & 0 \end{array}\right)\left(\begin{array}{l} \delta c_{k}\\ \delta u_{k} \end{array}\right)=\left(\begin{array}{l} \nabla L\left(c_{k},u_{k}\right)\\ {\left(h\left(c_{k}\right)\right)}^{T} \end{array}\right)$$

The corresponding parameters in Equation (22) can be denoted as:

(23) $$\nabla h\left(c_{k}\right)=2{\left[\begin{array}{ccc} c_{1,k} & c_{2,k} & c_{3,k} \end{array}\right]}^{T}$$

(24) $$\nabla L\left(c_{k},u_{k}\right)=\left[\begin{array}{c} 2\left(gk_{\text{axx}}+2g^{2}k_{2,x}c_{1,k}\right)\left(\mu_{1,k}-p_{x}^{k}\right)+2gk_{\text{ayx}}\left(\mu_{2,k}-p_{y}^{k}\right)+2gk_{\text{azx}}\left(\mu_{3,k}-p_{z}^{k}\right)+2u_{k}c_{1,k}\\ 2\left(gk_{\text{ayy}}+2g^{2}k_{2,y}c_{2,k}\right)\left(\mu_{2,k}-p_{y}^{k}\right)+2gk_{\text{azy}}\left(\mu_{3,k}-p_{z}^{k}\right)+2u_{k}c_{2,k}\\ 2\left(gk_{\text{azz}}+2g^{2}k_{2,z}c_{3,k}\right)\left(\mu_{3,k}-p_{z}^{k}\right)+2u_{k}c_{3,k} \end{array}\right]$$

(25) $$\begin{array}{l} \nabla_{c_{k}}^{2}L\left(c_{k},u_{k}\right)=\left[\begin{array}{ccc} l_{1,1} & l_{1,2} & l_{1,3}\\ l_{2,1} & l_{2,2} & l_{2,3}\\ l_{3,1} & l_{3,2} & l_{3,3} \end{array}\right]\\ l_{1,1}=2gk_{\text{axx}}\left(gk_{\text{axx}}+2g^{2}k_{2,x}c_{1,k}\right)+4g^{2}k_{2,x}\left(2gk_{\text{axx}}c_{1,k}+p_{0,x}+3g^{2}k_{2,x}c_{1,k}^{2}-p_{x}^{k}\right)+2g^{2}k_{\text{ayx}}^{2}+2g^{2}k_{\text{azx}}^{2}+2u_{k}\\ l_{1,2}=2gk_{\text{ayx}}\left(gk_{\text{ayy}}+2g^{2}k_{2,y}c_{2,k}\right)+2g^{2}k_{\text{azx}}k_{\text{azy}}\\ l_{1,3}=2gk_{\text{azx}}\left(gk_{\text{azz}}+2g^{2}k_{2,z}c_{3,k}\right)\\ l_{2,1}=2gk_{\text{ayx}}\left(gk_{\text{ayy}}+2g^{2}k_{2,y}c_{2,k}\right)+2g^{2}k_{\text{azy}}k_{\text{azx}}\\ l_{2,2}=2gk_{\text{ayy}}\left(gk_{\text{ayy}}+2g^{2}k_{2,y}c_{2,k}\right)+4g^{2}k_{2,y}\left(gk_{\text{ayx}}c_{1,k}+2gk_{\text{ayy}}c_{2,k}+p_{0,y}+3g^{2}k_{2,y}c_{2,k}^{2}-p_{y}^{k}\right)+2g^{2}k_{\text{azy}}^{2}+2u_{k}\\ l_{2,3}=2gk_{\text{azy}}\left(gk_{\text{azz}}+2g^{2}k_{2,z}c_{3,k}\right)\\ l_{3,1}=2gk_{\text{azx}}\left(gk_{\text{azz}}+2g^{2}k_{2,z}c_{3,k}\right)\\ l_{3,2}=2gk_{\text{azy}}\left(gk_{\text{azz}}+2g^{2}k_{2,z}c_{3,k}\right)\\ l_{3,3}=2gk_{\text{azz}}\left(gk_{\text{azz}}+2g^{2}k_{2,z}c_{3,k}\right)+4g^{2}k_{2,z}\left(gk_{\text{azx}}c_{1,k}+gk_{\text{azy}}c_{2,k}+2gk_{\text{azz}}c_{3,k}+p_{0,z}+3g^{2}k_{2,z}c_{3,k}^{2}-p_{z}^{k}\right)+2u_{k} \end{array}$$

Additionally, the initial Lagrange multiplier of *u_k,0_* can be chosen as a large integer such as 1,000.

#### 3.1.3. Flow of Two-Step Iterative Estimation

**Figure 1.**
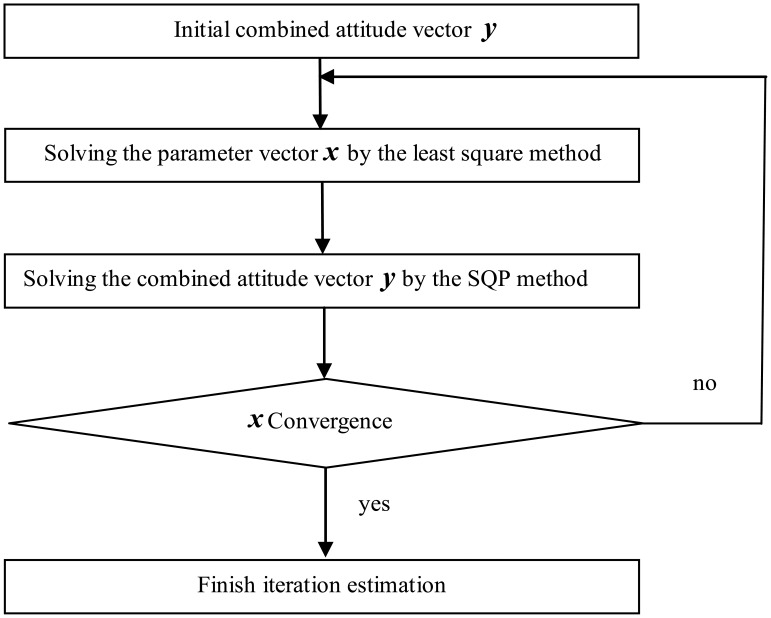
Flow of the two-step iterative estimation.

Consequently, the flow of two-step iterative estimation can be described in Figure 1 below:

The two-step iterative estimation method can also be used to estimate the parameters of the linear model of triaxial accelerometers.

### 3.2. Initial Values Selection of Two-Step Iterative Estimation

Refering to Figure 1, the initial values for the combined inclination vector in the two-step iterative estimation must be solved. Because the misalignments between *b*-frame and *a*-frame are small, the raw outputs of accelerometers in the gravitational field contain the rough inclination vector information. For example, the *x*-axis accelerometer attains the maximum raw output when the *x*-axis points upward, while the other two accelerometers approach the zero-value raw output. The raw output information coincides with the input specific force. The norm of raw outputs of triaxial accelerometers in the gravitational field is bounded by some lower bound and some upper bound. Thus an initial estimated inclination vector at the *k*-th position can be given as:

(26) $${\text{y}}_{k}=l_{k}^{b}=\left[\begin{array}{c} c_{1,k}\\ c_{2,k}\\ c_{3,k} \end{array}\right]\approx l_{k}^{a}=\frac{{\left[\begin{array}{ccc} p_{x}^{k} & p_{y}^{k} & p_{z}^{k} \end{array}\right]}^{T}}{\left\|{\left[\begin{array}{ccc} p_{x}^{k} & p_{y}^{k} & p_{z}^{k} \end{array}\right]}^{T}\right\|}$$

This initial value enables the two-step iterative algorithm and proves to be a very good candidate. It makes the parameters converge to the true values without exception in our simulations and experiments.

## 4. Scheme of Optimal Observations

Insufficient observations may degrade the calibration parameter accuracy. The optimal observation for estimating the calibration parameters should be analyzed. The fact that the norm of input specific force in the static case equals to the gravity value is a key to analyze the optimal observation scheme. As the sensitivity of the gravity value with respect to the calibration parameters depends on the observation positions, the maximum sensitivity principle can be utilized to get the optimal observation scheme, as done in [15]. According to Equations (3) and (6), the observation equation can be derived as follows:

(27) $$L={\left(\frac{p_{a,x}-p_{0,x}-k_{2,x}{\left(f_{x}^{b}\right)}^{2}-v_{a,x}}{k_{\text{axx}}}\right)}^{2}+{\left(\frac{p_{a,y}-p_{0,y}-k_{\text{ayx}}f_{x}^{b}-k_{2,y}{\left(f_{y}^{b}\right)}^{2}-v_{a,y}}{k_{\text{ayy}}}\right)}^{2}+{\left(\frac{p_{a,z}-p_{0,z}-k_{\text{azx}}f_{x}^{b}-k_{\text{azy}}f_{y}^{b}-k_{2,z}{\left(f_{z}^{b}\right)}^{2}-v_{a,z}}{k_{\text{azz}}}\right)}^{2}=g^{2}$$

Expanding Equation (27) results in the following equation:

(28) $$\begin{array}{l} L={\left(\frac{p_{a,x}-p_{0,x}-k_{2,x}{\left(f_{x}^{b}\right)}^{2}}{k_{\text{axx}}}\right)}^{2}-2v_{a,x}\frac{p_{a,x}-p_{0,x}-k_{2,x}{\left(f_{x}^{b}\right)}^{2}}{k_{\text{axx}}^{2}}+{\left(\frac{v_{a,x}}{k_{\text{axx}}}\right)}^{2}\\ +{\left(\frac{p_{a,y}-p_{0,y}-k_{\text{ayx}}f_{x}^{b}-k_{2,y}{\left(f_{y}^{b}\right)}^{2}}{k_{\text{ayy}}}\right)}^{2}-2v_{a,y}\frac{p_{a,y}-p_{0,y}-k_{\text{ayx}}f_{x}^{b}-k_{2,y}{\left(f_{y}^{b}\right)}^{2}}{k_{\text{ayy}}^{2}}+{\left(\frac{v_{a,y}}{k_{\text{ayy}}}\right)}^{2}\\ +{\left(\frac{p_{a,z}-p_{0,z}-k_{\text{azx}}f_{x}^{b}-k_{\text{azy}}f_{y}^{b}-k_{2,z}{\left(f_{z}^{b}\right)}^{2}}{k_{\text{azz}}}\right)}^{2}-2v_{a,z}\frac{p_{a,z}-p_{0,z}-k_{\text{azx}}f_{x}^{b}-k_{\text{azy}}f_{y}^{b}-k_{2,z}{\left(f_{z}^{b}\right)}^{2}}{k_{\text{azz}}^{2}}+{\left(\frac{v_{a,z}}{k_{\text{azz}}}\right)}^{2}=g^{2} \end{array}$$

The operator of mathematical expectation can be implemented on both sides of Equation (28) as below:

(29) $$\hat{L}={\left(\frac{{\hat{p}}_{a,x}-p_{0,x}-k_{2,x}{\left(f_{x}^{b}\right)}^{2}}{k_{\text{axx}}}\right)}^{2}+{\left(\frac{{\hat{p}}_{a,y}-p_{0,y}-k_{\text{ayx}}f_{x}^{b}-k_{2,y}{\left(f_{y}^{b}\right)}^{2}}{k_{\text{ayy}}}\right)}^{2}+{\left(\frac{{\hat{p}}_{a,z}-p_{0,z}-k_{\text{azx}}f_{x}^{b}-k_{\text{azy}}f_{y}^{b}-k_{2,z}{\left(f_{z}^{b}\right)}^{2}}{k_{\text{azz}}}\right)}^{2}=g^{2}-\Delta$$

The symbol of *p̂_a,i_* in Equation (29) denotes the expectation of *p̂_a,i_* and _Δ_ denotes the sum of measurement noise variance, *i.e.*, $\Delta =\frac{\sigma^{2}}{k_{\text{axx}}^{2}}+\frac{\sigma^{2}}{k_{\text{ayy}}^{2}}+\frac{\sigma^{2}}{k_{\text{azz}}^{2}}$.

Before the sensitivity analysis, the projection of the gravity vector in *b*-frame can be derived from Equation (6) as below:

(30) $$\left[\begin{array}{c} f_{x}^{b}\\ f_{y}^{b}\\ f_{z}^{b} \end{array}\right]=\left[\begin{array}{c} g\sin\theta \\ g\cos\theta \cos\phi \\ -g\cos\theta \sin\phi \end{array}\right],\theta \in \left[-\frac{\pi }{2},\frac{\pi }{2}\right],\phi \in \left[-\pi ,\pi \right]$$

According to Equations (29) and (30), we can get the sensitivity functions as shown in Appendix. By solving the maximum of the sensitivity functions, the optimal attitude angles can be obtained respectively as shown in Table 2.

In general, the optimal observations of the above 18 positions can be utilized to estimate the calibration parameters of triaxial accelerometers.

## 5. Simulation and Data Analysis

**Figure 2.**
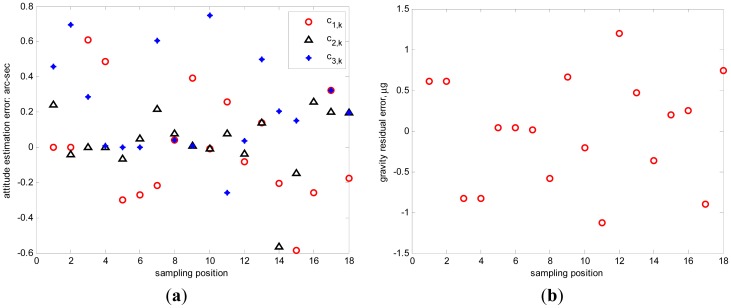
(**a**) The inclination vector estimation error; (**b**) The residual gravity error.

In the simulation settings, we assume that the measurement noise of accelerometers is $10\mu \text{g}/\sqrt{\text{Hz}}\left(1\sigma \right)$. The raw outputs of triaxial accelerometers (sampled at 100 Hz) are collected for 1 minute at each position. To diminish the measurement noise, the averaged raw output at each position is utilized to estimate the calibration parameters and inclination vectors. A set of calibrated results is shown in Table 3, as compared with the true calibration parameters. It shows that, the calibration error are less than 1 ppm for scale factors, less than 0.2 arc-seconds for misalignments, less than 2 μg for biases and less than 3 × 10^−6^ g/g^2^ for squared coefficients. Meanwhile, the estimation error of inclination vector is less than 1 arc-sec for 18 positions and the residual gravity error is less than 1.5 μg as shown in Figure 2(a,b), respectively.

**Figure 3.**
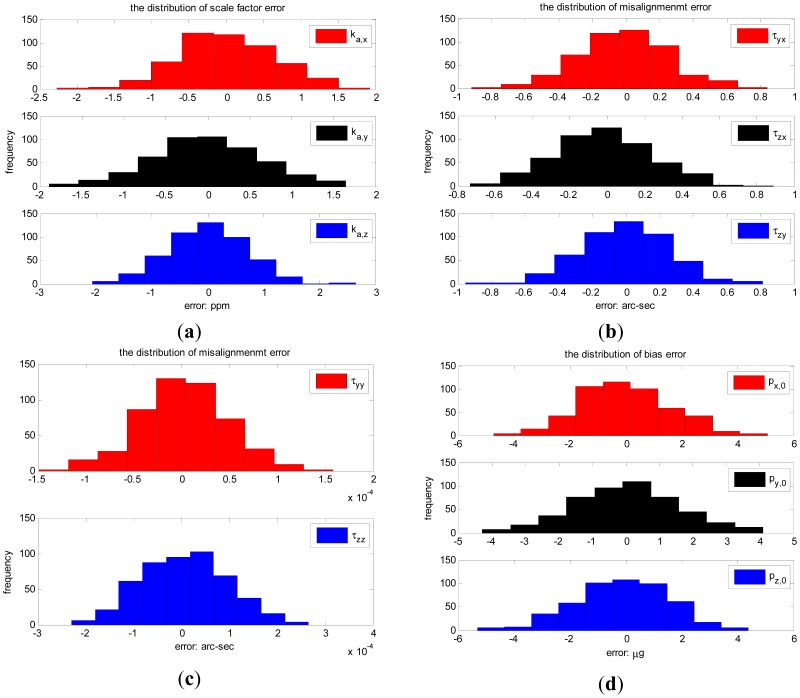

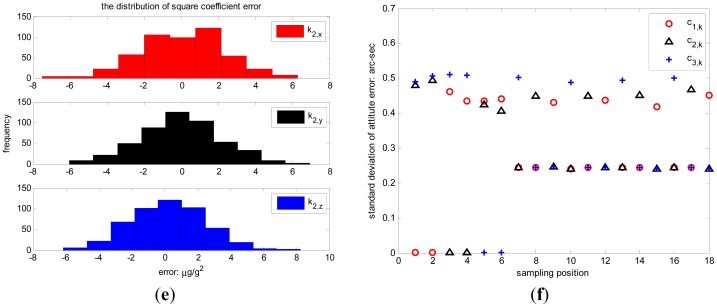
Error distributions: (**a**) scale factor errors. (**b**) the first set of misalignment errors. (**c**) the second set of misalignment errors. (**d**) bias errors. (**e**) squared coefficient errors. (**f**) inclination vector estimation errors.

The error distribution of calibration parameters in 500 Monte Carlo simulations are shown in Figure 3(a–e), associated with the standard deviation of inclination vector estimation error in Figure 3(f). The statistic errors of the calibration parameters are shown in Table 4.

The standard deviation for scale factor errors are less than 1 ppm, less than 0.3 arc-seconds for misalignment errors, less than 2 μg for bias errors and less than 3 × 10^−6^ g/g^2^ for squared coefficient errors. Meanwhile the standard deviation of inclination vector estimation error is less than 1 arc-sec for the optimal 18 observation positions.

## 6. Experiments and Data Analysis

**Figure 4.**
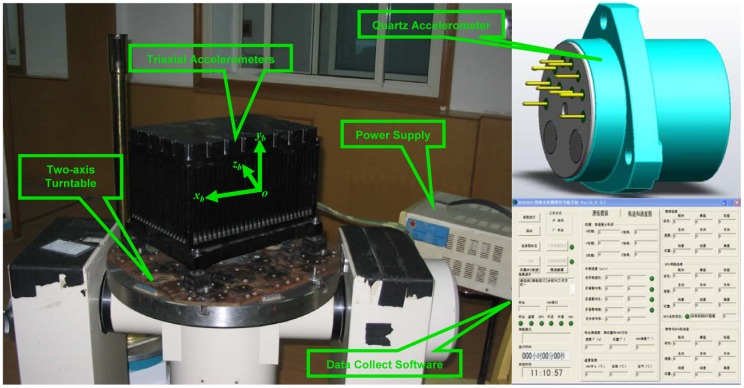
The experimental conditions for the calibration of triaxial accelerometers.

The calibration experiments are implemented for the triaxial quartz accelerometers with the noise of $10\mu \text{g}/\sqrt{\text{Hz}}$. A low-grade two-axis turntable with about 2 arc-minutes (1*σ*) is utilized to supply the approximately optimal 18-position static observation which also avoids the lever arms problem. However, the orientation information of the turntable is not used for estimation. The raw outputs of triaxial accelerometers are collected for 1 minute at each position with the sample frequency of 100 Hz. The averaged raw output at each position is utilized for the iterative estimation. The calibration procedure is implemented for three groups for comparison purpose. An experiment snapshot is given in Figure 4.

Three groups of calibration results for the same triaxial quartz accelerometers are shown in Table 5. The standard deviation of scale factor are less than 3 ppm, less than 0.1 arc-seconds for misalignments, less than 2 μg for biases and less than 2 × 10^−6^ g/g^2^ for squared coefficients. Obviously, the squared coefficient of *z*-accelerometer is larger than the other two accelerometers by one order of magnitude, so the squared coefficient of the *z*-accelerometer has more effect on the measurement accuracy of gravity value compared with the noise variance.

**Figure 5.**
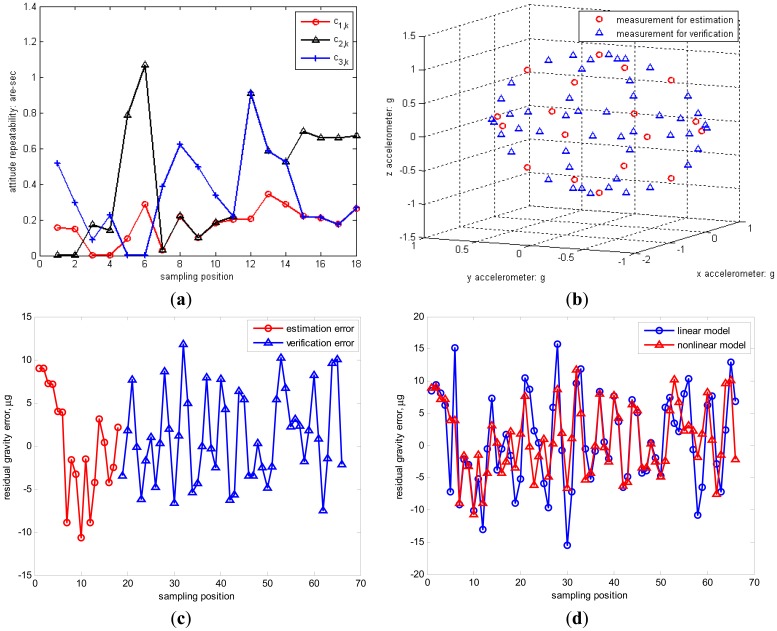
(**a**) The standard deviation of inclination vector estimation error; (**b**) The measurements for estimation and verification; (**c**) The estimation error and verification error; (**d**) Error comparison of linear and nonlinear models of triaxial accelerometers.

Meanwhile, the standard deviation of inclination vector estimation for 18 positions is less than 1 arc-second, as shown in Figure 5(a). To verify the measurement accuracy of triaxial accelerometers in the total gravity space, the positions for estimation and additional 48 positions for verification are respectively described in Figure 5(b) and the estimation and verification errors are shown in Figure 5(c). The standard deviation of estimated gravity error is 5.83 μg comparing with 5.24 μg for the verified gravity error. These two equivalent gravity errors validate the effectiveness of the chosen optimal 18-position observation. Thus the nonlinear model of triaxial accelerometers indicates enough accurate specific force measurement in the gravitational field. Besides, the calibration parameters of linear model are also estimated by the proposed two-step iterative method. The error comparison of linear and nonlinear models is shown in Figure 5(d). The standard deviation of estimation error by the linear model is 7.74 μg similar with 7.08 μg as the verified error. Consequently, the nonlinear model of triaxial accelerometers has higher accuracy than the linear model.

## 7. Conclusions

Laboratory calibration of triaxial accelerometers is a necessary step for high-accuracy specific force measurements. This paper proposes an improved iterative estimation method to derive the scale factors, misalignments, biases and squared coefficients associated with the inclination vectors at different positions. Additionally, no orientation information is required for the calibration. Thus the proposed calibration method is suitable for both low-grade and high-grade triaxial accelerometers. The main contributions of this paper can be summarized as follows:

1. Elements of direction cosine matrix are utilized for estimation instead of Euler angles to avoid the inclination vector computation singularities. The nonlinear errors of triaxial accelerometers are also estimated.
2. The initial inclination vectors are derived by normalizing raw outputs of triaxial accelerometers without any *a priori* information.
3. The optimal observation scheme is designed according to the maximum sensitivity principle to overcome the imperfections of models.

Simulation results illustrate the sufficient estimation accuracy of parameters and inclination vectors. The experiments have validated the effectiveness of optimal 18-position scheme by error comparison at estimated positions and verified positions. Comparison of the residual gravity error also proves that the measurement accuracy can be improved with the nonlinear model of triaxial quartz accelerometers with respect to the linear model case.

## Figures and Tables

**Table 1.** Definition of the related parameters.

**Parameters**	**Explanation**
*a*-frame	The non-orthogonal frame denoted by the accelerometers' sensitivity axes
*b*-frame	The orthogonal reference frame related to triaxial accelerometers
*n*-frame	The orthogonal local level frame
$C_{n}^{b}$	The direction cosine matrix from *n*-frame to *b*-frame
$T_{k}^{b}$	The non-orthogonal transformation from *b*-frame to *a*-frame
*p_i_*	The raw output of the *i*-axis accelerometer
*k_a,i_*	The scale factor of the *i*-axis accelerometer
τ*_yx_*,τ*_yy_* τ*_zx_*, τ*_zy_*, τ*_zz_*	The misalignments of triaxial accelerometers
*p_0,i_*	The bias of the *i*-axis accelerometer
*k*_2,i_	The squared coefficient of the *i*-axis accelerometer
*v*_a,i_	The measurement noise of the *i*-axis accelerometer
***f***^b^	The representation of the specific force in *b*-frame
***f****^2,b^*	The squared representation of the specific force in *b*-frame
$l_{b}^{a}$	The representation of unit gravity vector in *b*-frame at the *k*-th position

**Table 2.** The optimal observations for estimating calibration parameters.

**Estimated parameters**	**Optimal observations of (*θ,ϕ*)**			
*k_axx_,k_2,x_,p_0,x_*	$\left(\frac{\pi }{2},*\right)$		$\left(-\frac{\pi }{2},*\right)$	
k_ayy_,k_2,y_,p_0,y_	(0,0)		(0,π)	
k_azz_,k_2,z_,p_0,z_	$\left(0,\frac{\pi }{2}\right)$		$\left(0,-\frac{\pi }{2}\right)$	
*k_ayx_*	$\left(\frac{\pi }{4},0\right)$	$\left(-\frac{\pi }{4},0\right)$	$\left(\frac{\pi }{4},\pi \right)$	$\left(-\frac{\pi }{4},\pi \right)$
*k_azx_*	$\left(\frac{\pi }{4},\frac{\pi }{2}\right)$	$\left(\frac{\pi }{4},-\frac{\pi }{2}\right)$	$\left(-\frac{\pi }{4},\frac{\pi }{2}\right)$	$\left(-\frac{\pi }{4},-\frac{\pi }{2}\right)$
*k_azy_*	$\left(0,\frac{\pi }{4}\right)$	$\left(0,-\frac{\pi }{4}\right)$	$\left(0,\frac{3\pi }{4}\right)$	$\left(0,-\frac{3\pi }{4}\right)$

* Arbitrary values.

**Table 3.** A set of simulation results for triaxial accelerometers.

		**True parameters (pulse/m/s^2^)**	**Calibrated parameters (pulse/m/s^2^)**	**Error (ppm)**
**Scale factors**	*k_a,x_*	4,800	4,799.9992	−0.1601
	*k_a,y_*	4,900	4,899.9987	−0.2701
	*k_a,z_*	5,000	5,000.0051	1.0061
		**True parameters (rad)**	**Calibrated parameters (rad)**	**Error (arc-sec)**

**Misalignments**	*τ_yx_*	1.7453 × 10^−4^	1.7443 × 10^−4^	−0.0215
	*τ_zx_*	3.0229 × 10^−4^	3.0161 × 10^−4^	−0.1426
	*τ_zy_*	1.7453 × 10^−4^	1.7534 × 10^−4^	0.1672
	*τ_yy_*	0.99999998	0.99999998	3.7569 × 10^−6^
	*τ_zz_*	0.99999993	0.99999994	1.3787 × 10^−5^
		**True parameters (m/s^2^)**	**Calibrated parameters (m/s^2^)**	**Error (μg)**

**Biases**	*p_0,x_*	0.01	1.0016 × 10^−2^	1.6087
	*p_0,y_*	0.02	2.0002 × 10^−2^	0.3784
	*p_0,z_*	0.03	3.0007 × 10^−2^	1.4047
		**True parameters (g/g^2^)**	**Calibrated parameters (g/g^2^)**	**Error (g/g^2^)**

**Squared coefficients**	*k_2,x_*	1.0 × 10^−5^	1.0614 × 10^−5^	6.1459 × 10^−7^
	*k_2,y_*	2.0 × 10^−5^	2.2634 × 10^−5^	2.6347 × 10^−6^
	*k_2,x_*	3.0 × 10^−5^	3.0298 × 10^−5^	2.9839 × 10^−7^

**Table 4.** Calibration results of 500 Monte Carlo simulations for triaxial accelerometers.

		**Minimum**	**Maximum**	**Mean**	**Median**	**Deviation**
**Scale factor errors (ppm)**	*k_a,x_*	−2.273	1.924	0.007	−0.029	0.654
	*k_a,y_*	−1.896	1.644	−0.003	−0.022	0.647
	*k_a,z_*	−2.068	2.641	0.005	−0.001	0.709

**Misalignment errors (arc-sec)**	*τ_yx_*	−0.919	0.843	−0.015	−0.008	0.269
	*τ_zx_*	−0.736	0.889	−0.021	−0.029	0.257
	*τ_zy_*	-0.955	0.8127	−0.009	0.004	0.264
	*τ_yy_*	−1.488 × 10^−4^	1.584 × 10^−4^	2.39 × 10^−6^	1.41 × 10^−6^	4.698 × 10^−5^
	*τ_zz_*	−2.302 × 10^−4^	2.658 × 10^−4^	7.29 × 10^−6^	6.657 × 10^−6^	8.842 × 10^−5^

**Bias errors (**μg)	*p_0,x_*	−4.754	5.063	−0.021	−0.108	1.631
	*p_0,y_*	−4.295	4.097	0.017	0.014	1.576
	*p_0,z_*	−5.323	4.375	−0.318	−0.074	1.633

**Squared coefficient errors (g/g^2^)**	*k_2,x_*	−7.555 × 10^−6^	6.303 × 10^−6^	6.924 × 10^−8^	2.035 × 10^−7^	2.312 × 10^−6^
	*k_2,y_*	−6.057 × 10^−6^	6.938 × 10^−6^	−2.529 × 10^−9^	5.979 × 10^−8^	2.208 × 10^−6^
	*k_2,z_*	−6.185 × 10^−6^	8.245 × 10^−6^	2.043 × 10^−7^	2.079 × 10^−7^	2.271 × 10^−6^

**Table 5.** Three groups of calibration results for triaxial accelerometers.

		**#1 (pulse/m/s^2^)**	**#2 (pulse/m/s^2^)**	**#3 (pulse/m/s^2^)**	**Deviation (ppm)**
**Scale factors**	*k_a,x_*	5400.4641	5400.4518	5400.4338	2.8218
	*k_a,y_*	5233.0673	5233.0463	5233.0383	2.8621
	*k_a,z_*	5565.1729	5565.1577	5565.1492	2.1575
		**#1 (arc-sec)**	**#2 (arc-sec)**	**#3 (arc-sec)**	**Deviation (arc-sec)**

**Misalignments**	*τ_yx_*	−57.4562	−57.5347	−57.5613	0.0546
	*τ_zx_*	1.8155	1.7753	1.7188	0.0486
	*τ_zy_*	−9.0441	−9.0669	−9.1034	0.0299
	*τ_yy_*	206264.7982	206264.7982	206264.7982	0
	*τ_zz_*	206264.8061	206264.8061	206264.8061	0
		**#1 (μg)**	**#2 (μg)**	**#3 (μg)**	**Deviation (μg)**

**Biases**	*p_0,x_*	−19.9151	−18.5515	−19.2154	0.6819
	*p_0,y_*	1384.2615	1386.5858	1387.6277	1.7233
	*p_0,z_*	655.1133	656.8854	657.6365	1.2956
		**#1 (g/g^2^)**	**#2 (g/g^2^)**	**#3 (g/g^2^)**	**Deviation (g/g^2^)**

**Squared coefficients**	*k_2,x_*	2.9523 × 10^−6^	8.5401 × 10^−7^	1.0946 × 10^−6^	1.1483 × 10^−6^
	*k_2,y_*	−2.2722 × 10^−6^	−4.5485 × 10^−6^	−4.7544 × 10^−6^	1.3775 × 10^−6^
	*k_2,z_*	−3.3496 × 10^−5^	−3.5066 × 10^−5^	−3.6377 × 10^−5^	1.4424 × 10^−6^

## Appendix

The sensitivity functions can be derived from Equations (29) and (30) below:

(A-1) $$\frac{\partial \hat{L}}{\partial k_{\text{axx}}}=-2\frac{{\left({\hat{p}}_{a,x}-p_{0,x}-k_{2,x}{\left(f_{x}^{b}\right)}^{2}\right)}^{2}}{k_{\text{axx}}^{3}}=-\frac{2}{k_{\text{axx}}}{\left(f_{x}^{b}\right)}^{2}=-\frac{2g^{2}}{k_{\text{axx}}}{\left(\sin\theta \right)}^{2}$$

(A-2) $$\frac{\partial \hat{L}}{\partial p_{x,0}}=-2\frac{{\hat{p}}_{a,x}-p_{0,x}-k_{2,x}{\left(f_{x}^{b}\right)}^{2}}{k_{\text{axx}}^{2}}=-\frac{2}{k_{\text{axx}}}f_{x}^{b}=-\frac{2g}{k_{\text{axx}}}\sin\theta$$

(A-3) $$\frac{\partial \hat{L}}{\partial k_{\text{ayy}}}=-2\frac{{\left({\hat{p}}_{a,y}-p_{0,y}-k_{\text{ayx}}f_{x}^{b}-k_{2,y}{\left(f_{y}^{b}\right)}^{2}\right)}^{2}}{k_{\text{ayy}}^{3}}=-\frac{2}{k_{\text{ayy}}}{\left(f_{y}^{b}\right)}^{2}=-\frac{2g^{2}}{k_{\text{ayy}}}{\left(\cos\theta \cos\phi \right)}^{2}$$

(A-4) $$\frac{\partial \hat{L}}{\partial p_{y,0}}=-2\frac{{\hat{p}}_{a,y}-p_{0,y}-k_{\text{ayx}}f_{x}^{b}-k_{2,y}{\left(f_{y}^{b}\right)}^{2}}{k_{\text{ayy}}^{2}}=-\frac{2}{k_{\text{ayy}}}f_{y}^{b}=-\frac{2g}{k_{\text{ayy}}}\cos\theta \cos\phi$$

(A-5) $$\frac{\partial \hat{L}}{\partial k_{\text{ayx}}}=-2f_{x}^{b}\frac{{\hat{p}}_{a,y}-p_{0,y}-k_{\text{ayx}}f_{x}^{b}-k_{2,y}{\left(f_{y}^{b}\right)}^{2}}{k_{\text{ayy}}^{2}}=-\frac{2}{k_{\text{ayy}}}f_{x}^{b}f_{y}^{b}=-\frac{g^{2}}{k_{\text{ayy}}}\sin2\theta \cos\phi$$

(A-6) $$\frac{\partial \hat{L}}{\partial k_{\text{azz}}}=-2\frac{{\left({\hat{p}}_{a,z}-p_{0,z}-k_{\text{azx}}f_{x}^{b}-k_{\text{azy}}f_{y}^{b}-k_{2,z}{\left(f_{z}^{b}\right)}^{2}\right)}^{2}}{k_{\text{azz}}^{3}}=-\frac{2}{k_{\text{azz}}}{\left(f_{z}^{b}\right)}^{2}=-\frac{2g^{2}}{k_{\text{azz}}}{\left(\cos\theta \sin\phi \right)}^{2}$$

(A-7) $$\frac{\partial \hat{L}}{\partial k_{\text{azx}}}=-2f_{x}^{b}\frac{{\hat{p}}_{a,z}-p_{0,z}-k_{\text{azx}}f_{x}^{b}-k_{\text{azy}}f_{y}^{b}-k_{2,z}{\left(f_{z}^{b}\right)}^{2}}{k_{\text{azz}}^{2}}=-\frac{2}{k_{\text{azz}}}f_{x}^{b}f_{z}^{b}=\frac{g^{2}}{k_{\text{azz}}}\sin2\theta \sin\phi$$

(A-8) $$\frac{\partial \hat{L}}{\partial k_{\text{azy}}}=-2f_{y}^{b}\frac{{\hat{p}}_{a,z}-p_{0,z}-k_{\text{azx}}f_{x}^{b}-k_{\text{azy}}f_{y}^{b}-k_{2,z}{\left(f_{z}^{b}\right)}^{2}}{k_{\text{azz}}^{2}}=-\frac{2}{k_{\text{azz}}}f_{y}^{b}f_{z}^{b}=\frac{g^{2}}{k_{\text{azz}}}{\left(\cos\theta \right)}^{2}\sin2\phi$$

(A-9) $$\frac{\partial \hat{L}}{\partial p_{z,0}}=-2\frac{{\hat{p}}_{a,z}-p_{0,z}-k_{\text{azx}}f_{x}^{b}-k_{\text{azy}}f_{y}^{b}-k_{2,z}{\left(f_{z}^{b}\right)}^{2}}{k_{\text{azz}}^{2}}=-\frac{2}{k_{\text{azz}}}f_{z}^{b}=\frac{2}{k_{\text{azz}}}\cos\theta \sin\phi$$

(A-10) $$\frac{\partial \hat{L}}{\partial k_{2,x}}=-2{\left(f_{x}^{b}\right)}^{2}\frac{{\hat{p}}_{a,x}-p_{0,x}-k_{2,x}{\left(f_{x}^{b}\right)}^{2}}{k_{\text{axx}}^{2}}=-\frac{2}{k_{\text{axx}}}{\left(f_{x}^{b}\right)}^{3}=-\frac{2}{k_{\text{axx}}}{\left(\sin\theta \right)}^{3}$$

(A-11) $$\frac{\partial \hat{L}}{\partial k_{2,y}}=-2{\left(f_{y}^{b}\right)}^{2}\frac{{\hat{p}}_{a,y}-p_{0,y}-k_{\text{ayx}}f_{x}^{b}-k_{2,y}{\left(f_{y}^{b}\right)}^{2}}{k_{\text{ayy}}^{2}}=-\frac{2}{k_{\text{ayy}}}{\left(f_{y}^{b}\right)}^{3}=-\frac{2}{k_{\text{ayy}}}{\left(\cos\theta \cos\phi \right)}^{3}$$

(A-12) $$\frac{\partial \hat{L}}{\partial k_{2,z}}=-2{\left(f_{z}^{b}\right)}^{2}\frac{{\hat{p}}_{a,z}-p_{0,z}-k_{\text{azx}}f_{x}^{b}-k_{\text{azy}}f_{y}^{b}-k_{2,z}{\left(f_{z}^{b}\right)}^{2}}{k_{\text{azz}}^{2}}=-\frac{2}{k_{\text{azz}}}{\left(f_{z}^{b}\right)}^{3}=\frac{2}{k_{\text{azz}}}{\left(\cos\theta \sin\phi \right)}^{3}$$
